# Treatment preferences related to pulpectomy and prosthetic restorations among Lithuanian general dentists and prosthodontists

**DOI:** 10.2340/aos.v85.46336

**Published:** 2026-07-02

**Authors:** Viktorija Ašmankevičiūtė-Leminskė, Gediminas Žekonis

**Affiliations:** Lithuanian University of Health Sciences, Kaunas, Lithuania

**Keywords:** General dentists, prosthodontists, pulpectomy, prosthetic dentistry, restorations

## Abstract

**Objective:**

The objective of this study was to compare self-reported treatment preferences related to pulpectomy prior to prosthetic treatment, restoration selection for vital teeth, and impression-taking methods between Lithuanian general dentists (GDs) and prosthodontists (Ps).

**Material and methods:**

A questionnaire-based survey was conducted among 386 dentists in Lithuania, including GDs (62.4%) and Ps (37.6%). Data were analyzed using descriptive statistics and inferential tests (Chi-square and Fisher’s exact test), with a significance level of *p* < 0.05.

**Results:**

Most indications for pulpectomy were reported similarly by both groups; however, significant differences were observed for extensive coronal structure loss (> 50%) and deep caries, cervical lesions, gingival recession, short clinical crown, insufficient retention, enamel hypoplasia, and external root resorption (*p* < 0.05). Ps also more frequently reported using indirect restorations, including porcelain-fused-to-metal crowns, lithium disilicate ceramic inlays and onlays, and polymethyl methacrylate crowns. Conventional impressions were more common among GDs, whereas digital or combined workflows were more common among Ps.

**Conclusions:**

Lithuanian GDs and Ps reported broadly similar preferences regarding preservation of tooth vitality, but differed in selected pulpectomy indications, restorative options, and impression-taking methods. These differences may reflect variation in specialist training, clinical experience, and access to prosthetic workflows.

## Introduction

In Lithuania, after completing integrated dentistry studies, a dentist may practice as a general dentist (GD) and provide broad diagnostic, preventive, restorative, endodontic, periodontal, and basic prosthetic care. In contrast, to become prosthodontists (Ps), GDs must complete a 3-year-long residency program; therefore, prosthodontists have more advanced training in prosthetic treatment planning, indirect restorations, occlusion, laboratory-based workflows, and complex oral rehabilitation [1]. Both GDs and Ps can encounter similar clinical situations involving prosthetic rehabilitation of vital teeth; however, treatment approaches and preferences could be influenced by clinical experience, scope of practice, and differences in professional training.

Preservation of pulp vitality has become an increasingly important principle in contemporary dentistry. Current trends emphasize minimally invasive approaches and the use of conservative treatment methods whenever possible, reserving endodontic treatment for cases with clear indications [2–4]. Nevertheless, treatment preferences may vary among practitioners, particularly in borderline cases where treatment indications are not well defined.

Removal of the dental pulp may prevent the development of inflammatory processes following restoration placement and reduce the likelihood of needing to remove the prosthetic restoration for subsequent endodontic treatment. However, this approach is not without drawbacks, as endodontically treated teeth may become more fragile and are associated with loss of proprioception, structural changes, and an increased risk of complications [5, 6].

Patients seek dental treatment not only to restore function but also to achieve satisfactory aesthetic outcomes. In prosthetic dentistry, treatment planning is highly individualized and depends on both clinical findings and patient expectations. Dentists rely on their knowledge, experience, and available technologies to determine the most appropriate treatment approach for each case [7].

Advances in dental materials and techniques have significantly expanded the range of restorative options available. Modern prosthodontics offers a variety of solutions that allow clinicians to balance functional, biological, and aesthetic requirements. The selection of an appropriate restoration depends on multiple factors, including tooth vitality, the amount of remaining hard tissue, occlusal load, parafunctional habits, and aesthetic considerations, as well as the clinician’s skills and experience [8, 9].

Although specialist and generalist differences are expected, identifying the specific areas in which GDs and Ps differ is clinically relevant. Such information may help determine whether variation occurs mainly in expected areas, such as laboratory-based restorations and digital workflows, or also in biologically important decisions, such as pulpectomy of vital teeth before prosthetic treatment. In the Lithuanian context, data are limited regarding how GDs and Ps report approaching pulpectomy indications, restoration selection, and impression-taking methods within the same healthcare and educational environment.

Therefore, the aim of this study was to compare self-reported treatment preferences related to pulpectomy prior to prosthetic treatment, restoration selection for vital teeth, and impression-taking methods between Lithuanian GDs and Ps. By identifying variations in treatment preferences, this research aims to contribute to a better understanding of current practices and to highlight areas where greater consistency or further professional development may improve patient care outcomes.

## Materials and methods

### Study design

This cross-sectional study was approved by the Centre for Bioethics at the Lithuanian University of Health Sciences (No. BEC–OF–17). The study was reported in accordance with the CHERRIES (Checklist for Reporting Results of Internet E-Surveys) guidelines for online surveys.

A questionnaire was developed based on the study aims and relevant literature sources [10–20]. The questionnaire consisted of nine questions:

- Questions 1–4 (single-choice multiple-choice) collected general demographic and professional data.
- Question 5 (binary agree/disagree items) assessed indications for pulpectomy before prosthetic treatment.
- Questions 6–7 (single-choice multiple-choice) evaluated approaches to preserving tooth vitality.
- Question 8 (binary use/do-not-use items) assessed restoration choices for vital teeth.
- Question 9 (single-choice multiple-choice) evaluated impression-taking methods.

A total of 386 dentists participated in the study, including GDs and Ps.

Data were analyzed using Microsoft Excel, SPSS, and R software. Descriptive statistics were applied, and statistical significance was set at *p* < 0.05.

### Sampling and participants

Participants were recruited using a convenience sampling method. The questionnaire was distributed electronically via professional dental networks, social media groups, and direct communication channels commonly used by dentists in Lithuania. Participation in the study was voluntary and anonymous; thus, self-selection bias may have occurred, with dentists more interested in prosthodontic treatment or continuing education potentially being more likely to participate.

Eligible participants included licensed GDs and Ps currently practicing in Lithuania. Dentists not actively involved in clinical practice or those who did not complete the questionnaire in full were excluded from the analysis.

A total of 386 responses were collected. Incomplete or duplicate responses were excluded prior to analysis.

No formal *a priori* sample size calculation was performed because the study was exploratory and used convenience-based online recruitment. The final sample included 386 respondents, which was considered sufficient for descriptive analysis and exploratory comparison between GDs and Ps.

### Response rate

Due to the nature of online distribution through multiple channels, the exact number of dentists who received the questionnaire could not be determined; therefore, the response rate was not calculated. This represents a limitation of the study.

### Questionnaire development and validation

The questionnaire (Annex 1) was developed based on the study objectives and relevant literature sources [10–20]. It consisted of nine structured questions covering demographic information, pulpectomy indications, preservation of tooth vitality, restoration selection, and impression-taking methods.

The questionnaire was pilot tested among 10 practicing dentists to assess clarity and comprehensibility. Minor revisions were made accordingly. Because the questionnaire included independent thematic items rather than a unified psychometric construct, internal consistency analysis using Cronbach’s alpha was not considered appropriate. Nevertheless, the absence of formal external validation may be considered a limitation of the study.

### Data analysis

Data were analyzed using IBM SPSS Statistics and R. Descriptive statistics (frequencies and percentages) were used to summarize the data.

Comparisons between GDs and Ps were performed using the Chi-square (χ²) test of independence. When expected frequencies were less than five, Fisher’s exact test was applied. A significance level of *p* < 0.05 was considered statistically significant.

Cramér’s V was calculated as an exploratory effect size measure for statistically significant chi-square associations to describe the strength of association between the practitioner group and questionnaire responses. Effect sizes were interpreted descriptively and were not used as confirmatory evidence of clinical importance.

All statistical tests were two-tailed.

## Results

### General characteristics

A total of 386 dentists participated in the study, including GDs (*n* = 241) and Ps (*n* = 145). The distribution of respondents according to work experience, workplace, and methods of knowledge renewal is presented in Table 1.

**Table 1:** General characteristics of respondents.

Variable	GDs (*n* = 241) (%)	Ps (*n* = 145) (%)
Work experience (years):		
**< 5**	39.8	35.2
**5–10**	19.9	32.4
**10–15**	21.6	12.4
**> 15**	18.7	20.0
Workplace:		
**Private**	73.4	54.5
**Public**	12.0	0.0
**Both**	14.5	45.5
**Continuing education:**		
**Scientific articles**	2.9	17.2
**Courses/training**	71.0	63.4
**Colleagues**	13.7	8.3
**Knowledge sufficient**	12.4	11.0

GD: general dentist; P: prosthodontist.

**Table 2:** Indications for pulpectomy before prosthetic treatment.

Indication	GDs (%)	Ps (%)	*p*	Cramér’s *V*
Tooth sensitivity	40.7	47.6	0.187	—
Tooth pain	87.6	83.4	0.263	—
Percussion sensitivity	87.1	86.2	0.802	—
Extensive coronal structure loss (> 50%)/deep caries	61.4	42.8	< 0.001	0.18
Cervical lesions/recession	7.9	25.5	< 0.001	0.22
Periodontal pockets	31.5	25.5	0.202	—
Tooth wear (grade II)	20.7	22.8	0.630	—
Tooth wear (grade III)	63.1	63.4	0.953	—
Short crown/low retention	77.6	38.6	< 0.001	0.41
Patient preference	26.1	25.5	0.896	—
Crown discoloration	35.7	40.7	0.329	—
Enamel defects	13.7	37.2	< 0.001	0.26
External root resorption	73.4	85.5	0.003	0.15

GD: general dentist; P: prosthodontist.

*Cramér’s V is reported only for statistically significant associations (p < 0.05)*. Values represent the percentage of respondents who selected ‘agree’ that the listed condition was an indication for pulpectomy before prosthetic treatment.

**Table 3:** Restoration selection for vital teeth.

Restoration	GDs (%)	Ps (%)	*p*	Cramér’s *V*
**Ceramic veneers**	52.3	62.9	> 0.05	—
**Composite veneers**	26.2	25.7	> 0.05	—
**Pressed ceramic crowns**	66.2	85.7	0.036	0.11
**Zirconia crowns**	87.7	74.3	> 0.05	—
**PFM crowns**	40.7	78.6	< 0.001	0.30
**Lithium disilicate ceramic onlays**	35.3	57.9	< 0.001	0.27
**Lithium disilicate ceramic inlays**	31.5	61.4	< 0.001	0.28
**Temporary crowns**	55.4	51.4	> 0.05	—
**PMMA crowns**	30.8	74.3	< 0.001	0.32

GD: general dentist; P: prosthodontist; PFM: porcelain-fused-to-metal; PMMA: polymethyl. methacrylate.

*Cramér’s V is reported only for statistically significant associations (p < 0.05)*. Values represent the percentage of respondents who selected ‘use’ for each restorative option.

**Table 4:** Impression-taking methods.

Method	GDs (%)	Ps (%)
**Analog**	79.7	45.5
**Digital**	10.4	22.8
**Mixed**	10.0	31.7

GD: general dentist; P: prosthodontist.

*p* < 0.001, Cramér’s *V* = 0.35. Values represent respondents’ reported impression-taking method.

GDs more frequently worked exclusively in private practice, whereas Ps more often combined private and public practice. Continuing education was primarily achieved through courses and training in both groups, although Ps reported a higher tendency to engage with scientific literature.

### Indications for pulpectomy before prosthetic treatment

No statistically significant differences were observed between groups for several assessed indications. However, statistically significant differences were observed in several conditions, with varying effect sizes.

The strongest difference between groups was observed for **short clinical crown and insufficient retention** (*p* < 0.001, Cramér’s *V* = 0.41), indicating a strong association and differences in reported treatment preferences.

Moderate associations were found for:

- **Enamel hypoplasia or hypocalcification** (*p* < 0.001, *V* = 0.26)
- **Cervical lesions and gingival recession** (*p* < 0.001, *V* = 0.22).

These findings suggest statistically significant differences in how these conditions are interpreted between groups.

A smaller but statistically significant difference was observed for:

- **Extensive coronal structure loss (> 50%) and deep caries** (*p* < 0.001, *V* = 0.18).

This indicates a modest association between practitioner type and treatment preferences.

**External root resorption** also showed a statistically significant difference (*p* = 0.003, *V* = 0.15), although the effect size was weak, suggesting limited practical significance.

No statistically significant differences were found for tooth sensitivity, pain, percussion sensitivity, periodontal pockets, tooth wear, patient preference, or crown discoloration (*p* > 0.05).

### Preservation of tooth vitality

Most respondents in both groups reported attempting to preserve tooth vitality during prosthetic treatment (GDs: 69.2%, Ps: 54.3%), with no statistically significant difference (*p* = 0.283).

However, a statistically significant difference was observed in preparation strategy (*p* = 0.009), with GDs more frequently aiming to minimize the removal of hard tissue, while Ps more often considered individual tooth anatomy. The effect size for this difference was small (*V* ≈ 0.13), indicating limited practical significance despite statistical significance.

### Restoration selection

Significant differences were observed in the selection of several restorative options.

Moderate associations were identified for:

- **Porcelain-fused-to-metal (PFM) crowns** (*p* < 0.001, V ≈ 0.30)
- **Lithium disilicate ceramic inlays** (*p* < 0.001, V ≈ 0.28)
- **Lithium disilicate ceramic onlays** (*p* < 0.001, V ≈ 0.27)
- **Polymethyl methacrylate (PMMA) crowns** (*p* < 0.001, V ≈ 0.32).

These findings indicate statistically significant differences in restorative preferences between groups, with Ps more frequently selecting indirect and laboratory-fabricated restorations.

A smaller but statistically significant association was found for:

- **Pressed ceramic crowns** (*p* = 0.036, *V* ≈ 0.11).

No statistically significant differences were observed for ceramic veneers, composite veneers, zirconia crowns, or temporary restorations (*p* > 0.05).

### Impression-taking techniques

A statistically significant difference was observed in impression-taking methods between groups (*p* < 0.001), with a moderate effect size (*V* ≈ 0.35).

GDs predominantly used conventional (analog) impression techniques (79.7%), whereas Ps more frequently used digital (22.8%) or combined workflows (31.7%).

This indicates a statistically significant difference in reported impression-taking methods, with Ps reporting greater use of digital technologies.

## Discussion

### Pulpectomy indications

The present study demonstrates that GDs and Ps largely agree on most indications for pulpectomy prior to prosthetic treatment; however, significant differences were observed in specific clinical situations. These findings highlight variability in treatment preferences despite a shared theoretical background.

GDs more frequently reported recommending pulpectomy in cases of short clinical crown and insufficient retention. This finding may indicate a greater tendency toward preventive endodontic treatment prior to extensive prosthetic rehabilitation. In contrast, Ps may be more inclined to preserve tooth vitality and consider alternative restorative or prosthetic approaches aimed at improving retention while maintaining pulpal health. Current concepts in minimally invasive dentistry emphasize preservation of vital tooth structure whenever clinically feasible [2, 3, 10].

Differences were also observed in the management of cervical lesions, gingival recession, and enamel defects. Ps more frequently considered these conditions as potential indications for pulpectomy. Although such defects may be associated with dentin hypersensitivity, structural compromise, or restorative complexity [21–24], contemporary evidence generally supports conservative management unless irreversible pulpal pathology is present [10, 11]. These findings may reflect differences in the interpretation of borderline clinical situations and varying restorative philosophies between practitioner groups.

In the present study, GDs associated extensive coronal destruction and deep carious lesions with the need for pulpectomy more frequently than Ps did. This may reflect a more precautionary approach toward avoiding future endodontic complications after prosthetic rehabilitation. Previous studies have similarly reported concerns regarding overtreatment and variability in endodontic treatment recommendations among clinicians [6, 21].

External root resorption was more frequently considered an indication for pulpectomy by Ps. However, current evidence suggests that endodontic treatment is not always necessary in cases of external root resorption unless pulpal involvement or infection is present [25, 26]. This finding further highlights variability in the interpretation of pathological findings among clinicians.

Overall, the findings of the present study suggest that although both practitioner groups generally support preservation of tooth vitality, differences remain in the management of clinically ambiguous or borderline conditions. These differences may be associated with variations in specialist training, clinical experience, and prosthetic treatment philosophy.

### Restoration selection for vital teeth

The present study demonstrated significant differences in restoration selection between GDs and Ps. Ps reported greater preference for indirect restorations such as lithium disilicate ceramic inlays and onlays, PFM crowns, pressed ceramic crowns, and PMMA restorations.

The greater use of indirect restorations among Ps may reflect increased familiarity with laboratory-fabricated restorations, greater involvement in complex prosthetic rehabilitation, and more advanced prosthetic training. Previous studies have similarly shown that clinicians with specialist prosthodontic training are more likely to utilize indirect restorative techniques and advanced restorative materials [27–29].

Lithium disilicate ceramic inlays and onlays were more frequently selected by Ps in the present study. These restorations are often associated with more conservative preparation designs and preservation of healthy tooth structure compared to full-coverage crowns [30, 31]. Their increased use among prosthodontists may therefore reflect greater adoption of minimally invasive restorative concepts.

Significant differences were also observed in the use of PFM crowns and pressed ceramic crowns. Ps reported greater preference for these restorations, which may be related to broader experience with material selection, laboratory communication, and long-term prosthetic treatment planning. Although all-ceramic systems and zirconia restorations have gained popularity because of favorable aesthetic and mechanical properties [32–35], PFM crowns continue to demonstrate reliable long-term clinical performance [36, 37].

PMMA restorations were also used substantially more frequently by Ps. This finding may reflect more frequent use of provisional restorations during complex prosthetic rehabilitation procedures. PMMA materials remain widely used because of their favorable mechanical properties, ease of fabrication, and acceptable temporary aesthetic performance [38, 39].

Overall, these findings suggest that specialization and extensive prosthetic training may influence restorative preferences, particularly regarding indirect restorations.

### Impression-taking methods

The present study demonstrated statistically significant differences in impression-taking methods between GDs and Ps. GDs predominantly reported using conventional analog impression techniques, whereas Ps more frequently used digital or mixed workflows.

These findings may reflect differences in specialist training, access to digital technologies, frequency of complex prosthetic procedures, and integration of laboratory-based workflows into daily clinical practice. Previous studies have similarly demonstrated greater adoption of digital impression systems among prosthodontic specialists and clinicians performing advanced prosthetic rehabilitation [40–48].

Conventional elastomeric impressions remain widely used because of clinician familiarity, lower equipment costs, and broad accessibility [49–52]. However, digital impression systems offer several potential advantages, including improved patient comfort, simplified communication with dental laboratories, immediate visualization of preparations, and reduction of certain technical errors [40–42].

The greater use of digital workflows among Ps observed in the present study may also indicate increased engagement with contemporary digital prosthodontic concepts and CAD/CAM technologies. Nevertheless, adoption of digital systems may remain influenced by financial considerations, operator experience, and availability of digital infrastructure.

### Novelty and clinical relevance

The present study provides insight into self-reported prosthetic treatment preferences among Lithuanian GDs and Ps within the same healthcare and educational environment. Although differences between these groups are expected because of differences in training and scope of practice, the findings identify specific areas where reported preferences differ, particularly in selected pulpectomy indications, indirect restoration use, and impression-taking methods. These results may help identify topics for continuing education and may support future development of more standardized guidance for borderline prosthetic situations. However, because the study was based on self-reported questionnaire responses rather than standardized clinical scenarios, the findings should be interpreted as reported preferences rather than direct evidence of clinical behavior.

### Limitations

It is important to note the limitations of this study. The study was based on self-reported questionnaire responses rather than direct observation of clinical behavior, which may introduce response bias. In addition, convenience-based online recruitment may have resulted in self-selection bias and limited representativeness of the sample, as dentists with a greater interest in prosthodontics or continuing education may have been more likely to participate. Gender and other demographic variables were not collected, which limited further analysis of respondent characteristics. Because the survey was distributed through multiple online channels, the response rate could not be calculated. Furthermore, the questionnaire was not externally validated, which may limit the generalizability and methodological robustness of the findings.

### Clinical implications

The findings suggest that continuing education may be particularly useful in areas where reported preferences differed between GDs and Ps, including pulpectomy indications in borderline cases, selection of indirect restorations, and adoption of digital impression workflows. Future studies using standardized clinical vignettes are needed to determine whether these self-reported preferences correspond to actual clinical decision-making.

## Conclusions

Within the limitations of this cross-sectional survey, GDs and Ps demonstrated similar overall approaches toward preservation of tooth vitality during prosthetic treatment. However, statistically significant differences were observed in reported preferences regarding selected pulpectomy indications, restoration types, and impression-taking methods. Ps reported greater use of indirect restorations and digital workflows. These findings may reflect differences in specialist training and clinical experience.

## Supplementary Material



## Data Availability

The data supporting the findings of this study are available from the corresponding author upon reasonable request.

## Geolocation information

This study was conducted in Lithuania.
