# Supplementary material for: Treatment preferences related to pulpectomy and prosthetic restorations among Lithuanian general dentists and prosthodontists
Source: Acta Odontol Scand. 2026 Jul 2;85:46336. doi: 10.2340/aos.v85.46336 (PMC13338818; doi:10.2340/aos.v85.46336)
Supplement: Supplementary file 1 [file AOS-85-46336-s1.pdf]

Supplementary material has been published as submitted. It has not been copyedited or typeset by Acta Odontologica Scandinavica.

Annex 1. Questionnaire

QUESTIONNAIRE

I. General Questions

1. **Your qualification:**
   ☐ General dentist (GD)
   ☐ Prosthodontist (P)

2. **Years of practice:**
   ☐ 0–5 years
   ☐ 5–10 years
   ☐ 10–15 years
   ☐ More than 15 years
3. **Workplace:**
   ☐ Private sector
   ☐ Public sector
   ☐ Both public and private sectors
4. **How do you most commonly update your knowledge about endodontic treatment before prosthetic rehabilitation?**
   ☐ I regularly read scientific articles
   ☐ I attend courses and training programs
   ☐ I receive knowledge from colleagues
   ☐ My knowledge is sufficient based on my own clinical experience

II. Indications for pulpectomy

5. Indications for pulpectomy of vital teeth, based on your clinical experience

Statement	AGREE	DISAGREE
	(this is an indication)	*(this is not an indication)*
I. The tooth to be used as an abutment is sensitive to stimuli: cold, heat, or electric stimuli.	☐	☐
II. The tooth to be used as an abutment is painful.	☐	☐
III. The tooth to be used as an abutment is sensitive to vertical percussion.	☐	☐
IV. The crown of the tooth to be used as an abutment is severely damaged, with approximately 50% destruction and a deep carious lesion.	☐	☐

V. The cervical area of the tooth to be used as an abutment is exposed, showing gingival recession.	☐	☐
VI. The tooth to be used as an abutment has advanced periodontal recession, greater than 5 mm.	☐	☐
VII. The tooth to be used as an abutment has pathological Grade II crown mobility, corresponding to 1/3–2/3 loss of crown height.	☐	☐
VIII. The tooth to be used as an abutment has pathological Grade III crown mobility, corresponding to more than 2/3 loss of crown height.	☐	☐
IX. The crown of the tooth to be used as an abutment is short, and there is limited retention.	☐	☐
X. The patient requests endodontic treatment of the tooth before prosthetic rehabilitation.	☐	☐
XI. Discoloration changes are present in the crown of the tooth to be used as an abutment.	☐	☐
XII. Enamel hypoplasia or hypocalcification is present.	☐	☐
XIII. External root resorption is present in the tooth to be used as an abutment.	☐	☐

III. Possibilities of contemporary prosthetic rehabilitation of vital teeth

6. When preparing vital teeth for prosthetic rehabilitation, do you make every effort to preserve tooth vitality?

☐ Yes, this is very important to me
☐ Yes, but this depends on the clinical situation
☐ No, I strictly follow established indications
☐ I always treat all teeth endodontically before prosthetic rehabilitation

7. When preparing a vital tooth before restoration:

☐ I strictly follow the established preparation parameters
☐ I consider the individual anatomy of the tooth
☐ I try to preserve the hard tissues of the tooth as much as possible

8. To preserve tooth vitality during prosthetic rehabilitation, I use / do not use:

Material / restoration type	USE	DO NOT USE
Ceramic laminates	☐	☐
Composite resin laminates	☐	☐
Pressed ceramic crowns	☐	☐
Zirconia crowns	☐	☐

Metal-ceramic crowns	☐	☐
Lithium disilicate ceramic onlays	☐	☐
Lithium disilicate ceramic inlays	☐	☐
Thermoplastic temporary crowns	☐	☐
PMMA restorations	☐	☐
Lithium disilicate ceramic crowns	☐	☐
Glass-ceramic crowns	☐	☐

9. Which method do you use to take impressions for the fabrication of restorations?

☐ Analog method
☐ Digital method
☐ Both analog and digital methods
